# Improving knowledge of psychotropic prescribing in people with Intellectual Disability in primary care

**DOI:** 10.1371/journal.pone.0204178

**Published:** 2018-09-14

**Authors:** Rohit Shankar, Mike Wilcock

**Affiliations:** 1 Cornwall Partnership NHS Foundation Trust, Truro, United Kingdom; 2 University of Exeter Medical School, Exeter, United Kingdom; 3 Royal Cornwall Hospitals Trust, Truro, United Kingdom; Universita degli Studi di Perugia, ITALY

## Abstract

**Purpose:**

People with Intellectual disability (ID) are likely to be prescribed psychotropic medication particularly antipsychotics without a clear clinical indication. This has given rise to a national initiative in the UK to stop overprescribing medication in this vulnerable population. While the goals are simple it is unclear if specialist ID services or primary care services in the UK should look to lead. Further, it is uncertain if primary care practitioners (GPs) can be systematically educated of the latest good practice developments and concerns in this specialised area. This study surveyed the knowledge level of a sample of GPs in Cornwall UK (county of 538,000) post a structured tutorial on psychotropic medication and people with ID.

**Methods:**

A 21 item questionnaire was delivered in meetings organised for all the county GPs a year after a talk given to the same demographic. The questionnaire conducted an assessment of the knowledge of national guidance on use of psychotropic medication in ID based on the subjects covered in the tutorial.

**Results:**

Of the 60 expected GP participants the tutorial was attended by 44 GPs (73%) and the follow up meeting by 42 (70%). Ninety percent GPs in the follow up meeting filled the questionnaire. For 16 questions, more than 80% GPs gave correct responses whereas five questions attracted a correct answer from less than 80%. Majority of the GPs felt psychotropic medication management in people with ID should be specialist led.

**Conclusions:**

GPs’ knowledge of issues relevant to prescribing in people with ID benefitted from the tutorial. However a clear need for the psychotropic medication management to be delivered via specialist care emerged. This raises issues of resource allocation and debate on whether people with ID require specialist provision due to lack of ability in main stream primary care to manage their needs.

## Introduction

Intellectual disability (ID), also called Learning Disability, is a condition characterised by significant impairments of both intellectual and adaptive functioning, and an onset in early childhood, affecting about 1–2% of the general population [1]. The prevalence of people with Learning Disability (LD) in the UK is estimated to be around 1.4 million.

In the UK, people with ID tend to live either in family settings or in supported living arrangements. They may live with their biological families or in “Shared Lives” arrangements (i.e. other local families hosting them). People that do not live within a family setting may be supported by professional private sector care providers, funded by the local authority. Arrangements range from being in their own home with a professional care team, to reside in multi-occupancy residential homes. People with ID in the UK no longer live in large health institutions under the care of health providers like the National Health Service (NHS), which is the publicly funded national healthcare system for the UK. Direct care providers are generally not trained clinicians though some larger establishments may have a trained nurse to administer medication. Medical provision for community residential care is provided by the area primary care physician also called General Practitioner (GP). Where there are concerns of mental illness or serious behavioral disturbance a referral is made via the GP to specialist ID health teams. Specialist ID health teams have a range of multidisciplinary expertise including psychiatrists, psychologists, behavioral specialists, nurses, speech and language therapists, occupational therapists, dietian etc. They regularly work alongside social are workers. These professionals would look to understand the change in presentation of any person with ID on a bio-pycho-social model and decide on appropriate evidence based interventions and management strategies which could range from medication to recommendation of change in social setting to training of staff to support the concerned individual.

People with ID are amongst the most socially excluded and vulnerable groups, with greater health needs than the rest of the population [2]. Compared with their non-disabled peers, people with ID tend to have a lower life expectancy, and poorer health outcomes [2]. These included conditions such as asthma, epilepsy, hyperthyroidism, diabetes, severe mental illness, depression and dementia [3]. Of the adults with an ID, between 20% and 33% have autism and 22% co-morbid epilepsy [4]. Thus ID is associated with multiple morbidity, polypharmacy and sensory disabilities [4, 5]. The high rates of mental health comorbidity and physical health comorbidity contributes to significant premature mortality [6].

It is relatively common for people with ID to develop behaviours that challenge, and more common in people with more severe disability. It is estimated that 10% to 15% of people with ID have challenging behaviours [7, 8]. In this context, challenging behaviour has been described as behaviour which puts an individual or others at risk in any social situation and limits their access to services. National Institute for Health and Social Care Excellence guidance advises that specialists consider prescribing psychotropic medication (including antipsychotics, antidepressants, mood stabilisers and sedatives) to manage behaviour that challenges only in specific circumstances [9].

There is disproportionate prescribing of psychotropics in the ID population. From three years ago, 30,000 to 35,000 adults with ID nationally were likely to be prescribed an antidepressant or an antipsychotic without the key indications for doing so [10]. There is robust evidence that such prescribing could cause adverse physical health outcomes especially if not monitored regularly. NICE Guidelines have attempted to address the need for antipsychotics by suggesting regular check-ups to ensure that the medication is doing no harm [9].

Typically, multiple psychotropic drug use often starts at a specialist level which is then passed onto primary care with or without specialist follow up. Patients are likely to be discharged from specialist services once their referred health need is met. Hence, many GPs are overseeing the management and prescribing of these drugs long term. There is good prescribing practice guidance, aimed at primary and secondary healthcare clinicians, proposing standards for improving clinical practice in this area [11], and the main aim of the NHS England STOMP (Stopping Over-Medication of People with a Learning Disability) campaign [12] to support the safe and appropriate use of antipsychotic medicines[13]. However as yet there is no recognised structured mechanism, pathway or guidance to deliver STOMP practically into primary care in particular.

Though some aspects of general practice management of patients with ID have been reported in the literature, [14, 15, and 16] there is little published research that focuses on prescribing competence and knowledge of general practitioners.

The aim of this study was to survey the knowledge level of a sample of GPs in one Clinical Commissioning Group (CCG) in relation to the use of psychotropic medication in ID. CCGs were created following the Health and Social Care Act in 2012 in the UK. They are clinically-led statutory NHS bodies responsible for the planning and commissioning of health care services for their local area. For Cornwall a county of the UK there is one CCG which covers the population of 538,000.

## Methods

Across Cornwall, locality-based prescribing meetings are held in the north, central and west locality areas of the CCG four times a year. These twelve meetings a year, organised by NHS Cornwall CCG Medicines Optimisation Team, are intended to have a focus on clinical prescribing and medicines optimisation. A GP prescribing lead from each primary care practice is invited to attend these meetings and disseminate the learning within their own practice.

A structured one hour talk was designed taking into account current concerns of prescribing in people with ID, national guidance, local issues, audits and challenges. The talk was developed by a co-author (RS) and consisted of all relevant good practice and national context on this subject since 2013 including all relevant NHS England publications. Further local audits and data were co-located next to national context. The same talk was delivered to the audiences of the three different localities in the winter of 2016. It was ascertained that the participants had no or minimal knowledge of the issue presented. This was identified by the coordinator of the meetings. Further there were no GPs with a recognised extended role or special interest in ID or mental health (GPSIs). In addition the questions/comments made at the presentation was identifiable with the cohort being typical of generalist GPs. In winter 2017 at each of the three locality meetings, 12 months after the presentation the GPs were asked to complete a questionnaire with 21 questions. The questionnaire was constructed by the authors and consultation was had with a GP to ensure it was pitched at the ‘right level’. No demographic details were asked for and the survey was anonymous. The questionnaire contained questions assessing knowledge on ID and on prescribing in ID. This small survey consisted of questions with predetermined answers requiring true / false replies, and one question that allowed for free text comments. The questionnaire followed main points of the presentation. A total correct reply score of 80% was deemed to be a reasonable threshold to use. Analysis of data was performed using Microsoft Excel. Descriptive statistics were used to describe and summarise the data highlighting the main elements of the study. Majority of the attendees to the talk were attendees to the survey. Even if the same GP who attended the talk was not present it was expected that the information of the talk would have been shared in the primary care practice the GP represented thus ensuring in theory that all GPs in Cornwall would have had an education that GPs would need to be aware of safe and appropriate use of psychotropic drugs, particularly antipsychotics and is a priority for people with ID. This sharing of information was supported by the CCG Prescribing Team email communication to practices summarising the key points of the talk and signposting to the STOMP presentation.

### Ethics

No ethical permission was required as this was a survey to evaluate training. Further it was to a group on medical practitioners where consent was implicit by returning the survey form. All participants were advised at the start of the survey that participation was voluntary and their replies i.e. data would be anonymised and analysed. We also used the NHS Health research authority tool (http://www.hra-decisiontools.org.uk/research/index.html) which helped confirm that no ethics is needed for this project (S1 File)

## Results

There are 60 GP surgeries (primary care centres) within NHS Cornwall CCG. The winter 2016 talk was attended by 44 GPs (73% attendance) across the three meetings. In winter 2017 the three meetings were attended by a total of 42 GPs (70% attendance), with completed questionnaires returned from 38 (90.5%). No other GP characteristics were recorded. As regards knowledge of associated co-morbidities, the respondents, in the main, correctly answered 4 out of the 5 questions posed (Table 1). It was only the association between ID and substance misuse that was poorly recognised with less than half giving the correct response. The respondents appeared more knowledgeable when asked about possible common health problems associated with challenging behaviour in people with ID (Table 2). When questioned about their knowledge of relevant NICE guidelines, the percentage of GPs providing correct responses were high (in excess of 80%) apart from one of the questions about when antipsychotics should be used (Table 3). Similarly, two of the 5 questions about NICE guidelines and action to be taken for patients taking antipsychotics identified gaps in knowledge with correct responses received by less than 80% of GPs (Table 4). Responses to a question about GPs views on who is responsible (specialist or GP) for certain elements of psychotropic drug prescribing are shown in Table 5.

**Table 1:** True or false questions about GPs perception of people with ID.

	correct answer	Correct responses N (%)
Less likely than the general population to develop schizophrenia	False	32 (84.2%)
Less likely than the general population to develop depression	False	37 (97.4%)
More likely than the general population to develop major mental illnesses	True	33 (86.8%)
Less likely than the general population to have epilepsy	False	34 (89.5%)
Less likely than the general population to develop substance misuse disorders	False	18 (47.4%)

**Table 2:** True or false questions about which of these common physical health concerns could predispose to challenging behaviour in people with ID.

	correct answer	Correct responses N (%)
Constipation	True	38 (100%)
Severe ear ache	True	37 (97.4%)
Depression	True	37 (97.4%)
Bereavement	True	36 (94.7%)
Dementia	True	38 (100%)

**Table 3:** True or false questions based on the NICE guidelines for the treatment of challenging behaviour in ID.

	correct answer	Correct responses N (%)
Rule out the use of antipsychotic medication and off label prescribing	False	35 (92.1%)
Suggest that antipsychotics should be used only if psychological/ other interventions do not produce change	True	26 (68.4%)
Suggests that antipsychotics should be used only in combination with psychological/ other interventions	True	33 (86.8%)
Suggests that antipsychotics can be used if the risk to the person or others is very severe	True	33 (86.8%)
Recommend that antipsychotics should be initially prescribed by a specialist	True	37 (97.4%)

**Table 4:** True or false questions based on the NICE guidelines for the treatment of mental health problems in ID on antipsychotic prescribing.

	correct answer	Correct responses N (%)
Consider reducing or discontinuing antipsychotics	True	38 (100%)
Consider referral to a psychiatrist experienced in working with people with learning disabilities and mental health problems	True	36 (94.7%)
Document, every 3 months, the reasons for continuing the prescription if it is not reduced or discontinued	False	18 (47.4%)
Document annually the reasons for continuing the prescription if it is not reduced or discontinued	True	27 (71.1%)
Review the condition after reducing or discontinuing a prescription	True	38 (100%)

**Table 5:** Question on whether specialists or GPs should be responsible for ensuring good practice standards for psychotropic drug prescribing in people with ID.

	Specialist	GP	Either	Both
The indication(s) and rationale for prescribing the psychotropic drug should be clearly stated, including whether the prescribing is off-label, polypharmacy or high dose.	26	0	1	11
Consent-to-treatment procedures (or best interest’s decision-making processes) should be followed and documented.	17	0	3	18
There should be regular monitoring of treatment response and side-effects (preferably every 3 months or less, at a minimum every 6 months).	16	1	6	15
Review and evaluation of the need for continuation or discontinuation of the psychotropic drug should be undertaken on a regular basis (preferably every 3 months or less, at a minimum every 6 months) or whenever there is a request from patients, carers or other professionals.	19	1	7	11

When asked on a scale of 1 to 5, where 1 is not a priority at all and 5 is very important, how much of a priority the national programme to stop overmedication of people with ID was to the respondent and their practice colleagues, the mean score was 2.9, median three (range 1–5) and mode 3 (n = 15). In response to a question asking if their practice would be interested in taking part in an initiative for GP practices, community pharmacists, and specialist ID mental health teams to systematically stratify and reduce the level of antipsychotic prescribing, 24 replied ‘yes’, 12 ‘maybe’ and two ‘no’.

Free text comments on this subject of ID and prescribing were made by only four GPs with three of these highlighting the importance of resourcing a shared care approach to prescribing and monitoring of antipsychotics, and one GP questioning whether a specialist can fully understand the needs of patient with ID and their carer.

## Discussion

This small study looked to understand two issues. First, can the knowledge of GPs on complex subjects such as ID and psychotropic prescribing be increased and retained by them using a structured talk by a specialist. Secondly, having been informed then what the general perception towards priority and management by GPs towards this complex and vulnerable group of people with ID and their management of mental disorders and/or challenging behaviours is.

The results have shown that a large sample of GPs in the locality subsequent to the talk appear to have a reasonable knowledge of ID and various aspects of good practice prescribing. The areas where correct responses were less than 80% related to knowledge of the association between ID and substance misuse, and knowledge of two recommendations from NICE guidelines that antipsychotics should be used only if psychological or other interventions do not produce change and the frequency of documentation of reasons for continuing an antipsychotic prescription if it is not reduced or discontinued.

Inappropriate prescribing and monitoring of side effects of antipsychotic in ID has been well described [17, 18]. Whether this can be categorised solely as a specialist problem or a primary care problem or a shared care issue is controversial and not necessarily helpful but been a subject of significant debate. One of the resources associated with the national campaign urged GPs to undertake a number of steps, including appointing a GP lead, obtaining details of all people with an ID on psychotropic medication, and checking if attempts at drug reduction or withdrawal had already been made. To what extent this has occurred in general practice is unclear, and challenges to bringing about the necessary change, even in the setting of a randomised controlled trial, have been described [18, 19, 20].

Our study continues to highlight the division on opinion on how care needs to be delivered to this complex vulnerable population. The opinion generally is towards it being a specialist role though a significant minority are willing to consider a shared care arrangement. There is no interest in primary care to support people with ID by themselves. It is pleasing that 24 GPs expressed an interest in taking part in an initiative to systematically stratify and reduce the level of antipsychotic prescribing and this is underway across Cornwall [18]. It was evident in our survey that even the GPs who showed good retention on the subject had some reservations in taking on this work. This could be due to a myriad of reasons which include issues of complexity, lack of significant numbers to justify resources to such a specialist need and time. It was recognisable that the situation sits uncomfortably between specialist and primary care without possibly clear ownership. To help reduce these interface tensions we would propose a concept of a STOMP practitioner who sits between primary and secondary care. The role of the individual who could be a nurse prescriber or a pharmacist is to screen GP caseloads and stratify cases based on complexity and need and provide continued education to primary care and patients. This would help rationalise resources at both primary and secondary care appropriately and ensure the most vulnerable are prioritised thus not being an undue burden on specialist resources while equally not being an encumbrance to primary care. Resource implications for the role of such a person should be minimal.

### Strengths and limitations

We recognise the limitations with this small study undertaken in just one CCG with a self-selected group of GPs. We had no exact record of which responding GPs attended the educational presentation 12 months earlier, though it is believed that the majority (at least three-quarters) would have done so. As we did not examine pre-awareness to the topic there might have been the theoretical possibility of some GPs who due to personal experience (e.g. relative with ID) or professional interest have a higher level of knowledge. We also used a survey which could have introduced biases such as recall bias and answering tendencies [21]. Strengths recognised that were the presence of over 70% of GPs in both the meeting cycles and 90% response rate of the survey.

## Conclusion

It is encouraging that, in the main, respondents appeared to demonstrate good retention and knowledge of ID and relevant NICE guidelines post training. It is recommended that this model of communication be considered as part of the initiative to reduce the burden of psychotropics in people with ID in particular but also other special populations such as the elderly in general. We recognise the complexities other areas might have in adopting an equivalent frame work. However we are mindful that a consistent evidence based message delivered from the expert service to a target audience of GPs inscribing the national picture and moulding it to local need is both informative and educational thus local GP networks and CPDs need be targeted.

## Supporting information

S1 File Result—NOT Research.pdf—Evidence for not requiring formal ethics. (PDF)
